# Radiation Outcome in Mechanical Thrombectomy of Acute Ischemic Stroke

**DOI:** 10.1515/tnsci-2019-0002

**Published:** 2019-04-25

**Authors:** Xiaoying Cai, Xianhui Ding, Wenbin Wang, Ke Yang, Zhiming Zhou, Yannan Fang, XiaoLei Shi

**Affiliations:** 1 Department of Anesthesiology, The First Affiliated Hospital of Sun Yat-sen University, Guangzhou 518000, China; 2 Guangdong Key Laboratory for Diagnosis and Treatment of Major Neurological Diseases, Department of Neurology, National Key Clinical Department and Key Discipline of Neurology, The First Affiliated Hospital, Sun Yat-sen University, Guangzhou, China; 3 Department of Neurology, The First Affiliated Hospital, Yijishan Hospital of Wannan Medical College, Wuhu 241000, China

**Keywords:** occlusion, stroke, mechanical thrombectomy

## Abstract

**Objective:**

Mechanical thrombectomy is recommended for acute ischemic stroke (AIS) with large artery occlusion. Radiation during the endovascular procedure would increase the risk of skin diseases. We sought to identify radiation outcomes during mechanical thrombectomy.

**Methodology:**

We prospectively collected and analyzed radiation parameters during mechanical thrombectomy in 41 patients affected with acute cerebral artery occlusion.

**Results:**

There were 41 cases (68.73 ± 11.05 years) in this study, with a National Institute Health Stroke Scale (NIHSS) score of 15.66 ± 5.94. The time parameters were recorded as following: 84.45 ± 31.66 min (operation duration), 129.71 ± 81.14 s (angiographic run), 16.02 ± 11.03 min (fluoroscopy) and 18.19 ± 11.14 min (angiographic exposure). The doses produced in the procedure were: 1276.43 ± 1647.56 mGy (shot dose), 607.26 ± 412.34 mGy (fluoroscopy) and 1635.52 ± 593.65 mGy (angiographic exposure). Further analysis discovered no association between NIHSS and these time and radiation parameters (P > 0.05).

**Conclusion:**

This study provided the description of radiation details during mechanical thrombectomy for acute cerebral artery occlusion. The stroke severity would not influence the procedure parameters.

## Introduction

Ischemic stroke presents a heavy economic and health burden on the population. It usually causes a series of neurological deficits, disability and mortality. Intravenous thrombolysis is initially used for the disease in acute stage. [1] With the improvement of device technology and the understanding of this disease, endovascular therapy is strongly recommended for acute ischemic stroke (AIS) within time window. [2,3] Mechanical thrombectomy is preferred for large vessel occlusion, according to the current evidences. [4] However, radiation produced during the X-ray assisted procedure is an unwanted incidence, which would increase the risk of skin and malignant diseases. [5,6] And currently, limited studies are available to detect the radiation effects during mechanical thrombectomy for acute stroke patients. The objective of the study is to provide a description of radiation details during mechanical thrombectomy.

## Materials and methods

### Participants

The study was approved by the institutional review board of our hospital. Investigations involving humans was performed in accordance with the principles of Declaration of Helsinki. We retrospectively analyzed the prospectively collected data of subjects with AIS who were treated between November, 2017 and March, 2018. The definition of AIS was done based upon the World Health Organization Monitoring Trends and Determinants in Cardiovascular Disease project. [7] Patients fulfilling the following criteria were included in this study: 1) ≥ 18 years old; 2) stroke affected for the first time; 3) neurological deficits within 6 hours from symptom onset; 4) ischemic lesions confirmed by magnetic resonance imaging (MRI) or no hemorrhage detected by computed tomography (CT) at admission; 5) no contraindications for endovascular treatme; 6) written consent form from patients or their guardians.

### National Institute Health Stroke Scale (NIHSS) assessment

All the subjects received NIHSS assessment at admission by two senior physicians in Department of Neurology, respectively. If any disagreement existed, a final score was calculated through consensus-based discussion and decided by the senior author.

### Interventional procedure

All procedures were performed by senior interventional neuroradiologists using biplane angiography unit with 3D rotational angiography capability (SIEMENS Artis Q zeego) under general anesthesia or conscious sedation. For endovascular access, an 8F sheath was placed in the right femoral artery via a standard approach. Then, an 8F catheter was introduced to the arch of aorta under fluoroscopy and navigated to the common carotid artery of the occluded side. In those with occlusion in basilar artery, the catheter was placed into the dominant or most navigable vertebral artery. For the occlusion with vertebral artery, the catheter was advanced into the subclavian artery. Then, a microcatheter tip is negotiated into the distal part of the thrombus, and the Solitaire stent-retriver device was advanced through the microcatheter. Then the stent-retriver device was deployed by pulling back the microcatherter. Device and microcatheter were simultaneously pulled out under continuous aspiration through the intermediate catheter, which was applied with a 60ml syringe. Angiographic runs were performed to control flow restoration. Angiographic runs were repeated to record the results. Thrombectomy was done again when no sufficient recanalization was not achieved. And final recanalization was tested according to Thrombolysis in Cerebral Infarction (TICI) classification, [8] with the definition of good recanalization as 2b and 3.

### Data Collection

Baseline data were gathered including age, gender, medical history (hypertension, diabetes mellitus, arterial fibrillation, hyperglycemia, arch type), NIHSS at admission and occluded artery. Time parameter included operation duration, angiographic run and fluoroscopy time. Operation time was defined from femoral artery puncture to mechanical closure or manual compression for the puncture site. Angiographic run time was the accumulated time of angiographic shots of contrast medium. Fluoroscopy time meant the time cost to place catheter, guide wire and stent-retriver devices into suitable sites. Radiation parameter were analyzed, including kerma area product (KAP), angiographic run dose, fluoroscopy dose and cumulative dose. KAP was defined as product of the kerma and the area exposed to radiation. Angiographic run dose meant the radiation dose generated in all angiographic runs. Fluoroscopy dose was defined as the dose during the operator adjust catheter, guide wire and other devices until a good recanalization was achieved. Cumulative dose was concluded by adding angiographic run dose and fluoroscopy dose together.

### Statistical analysis

Demographic, time and radiation variables were displayed. Continuous data were depicted by mean and standard deviation. Correlations were determined using the Spearman’s correlation coefficient between NIHSS at admission and radiation as well as time parameters. All significance tests were 2 sided, and *P* < 0.05 were considered statistically significant. All analyses were performed using SPSS 19.0 software.

## Results

### Patient characteristics

The baseline characteristics were shown in Table 1. A total of 41 cases received mechanical thrombectomy, with 23 men and 18 women. The average age was 68.73 ± 11.05 years old. The previous history was described as: 13 with smoking, 12 with alcohol use, 27 with hypertension, 10 with diabetes, 27 with arterial fibrillation and 12 with hyperglycemia. An average NIHSS score of 15.66 ± 5.94 was calculated among them. For occlusion site analysis (Table 2), 11 patients suffered internal carotid artery (ICA) occlusion. 27 middle cerebral artery (MCA)s and 3 posterior arteries were affected.

**Table 1:** Baseline information of the study. y, year; HT, hypertension; DM, diabetes mellitus; AF, arterial fibrillation; NIHSS, National Institute Health Stroke Scale.

Variables	All
No.	41
Age (y)	68.73 ± 11.05
Male	23
Smoke	13
Alcohol	12
HT	27
DM	10
AF	27
Hyperglycemia	12
Arch type	
Ⅰ	12
Ⅱ	19
Ⅲ	10
NIHSS	15.66 ± 5.94

**Table 2:** Endovascular treatment details of the participants. ICA, internal carotid artery; MCA, middle cerebral artery.

Variable	No.
ICA	11
MCA	27
Posterior circulation	3

### Procedural parameters

The time and radiation parameters of the subjects were shown in Table 3. These patients had an operation duration of 84.45 ± 31.66 min, with angiographic run of 129.71 ± 81.14 s and fluoroscopy time of 16.02 ± 11.03 min. KAP during the procedure was 26519.68 ± 9502.81 Gy-cm^2^. For radiation doses, angiographic run induced a dose of 1276.43 ± 1647.56 mGy. And fluoroscopy caused 607.26 ± 412.34 mGy exposure. The cumulative dose generated was 1635.52 ± 593.65 mGy.

**Table 3:** Endovascular treatment details of all patients. min, minute; s, second; ICA, internal carotid artery; MCA, middle cerebral artery; KAP, kerma area product.

		Time			Dose		

Variable	Operation duration (min)	Angiographic run (s)	Fluoroscopy (min)	KAP (Gy- cm2)	Angiographic run (mGy)	Fluoroscopy (mGy)	Cumulative (mGy)
All	84.45 ± 31.66	129.71 ± 81.14	16.02 ± 11.03	26519.68 ± 9502.81	1276.43 ± 1647.56	607.26 ± 412.34	1635.52 ± 593.65
Site							
ICA	79.95 ± 14.43	151.93 ± 127.46	13.28 ± 4.01	23828.00 ± 7348.11	1969.34 ± 3119.98	477.63 ± 180.10	1520.69 ± 311.00
MCA	84.29 ± 34.58	125.73 ± 58.37	15.19 ± 10.21	26279.70 ±8662.80	1031.08 ± 370.60	585.35 ± 351.24	1616.95 ± 614.17
Posterior	102.36 ± 53.59	83.99 ± 8.56	33.59 ± 22.04	38549.00 ± 17402.26	943.93 ± 461.55	1279.73 ± 925.88	2223.67 ± 1032.03

### Analysis of procedural parameters

We analyzed the association between age, NIHSS and these parameters, and found that age and NIHSS score were not correlated with these values (*P* > 0.05) (Table 4). Then the procedural parameters were examined based on gender, arch type, smoking, alcohol use, hypertension, diabetes, arterial fibrillation and hyperglycemia. And no differences were revealed.

**Table 4:** The association between NIHSS and endovascular procedure parameters. KAP, kerma area product.

	P_1_	P_2_
**Time**		
Operation duration (min)	0.487	0.671
Angiographic run (s)	0.109	0.919
Fluoroscopy (min)	0.494	0.707
KAP (Gy-cm^2^)	0.822	0.883
**Radiation dose**		
Angiographic run (mGy)	0.398	0.085
Fluoroscopy (mGy)	0.653	0.607
Cumulative (mGy)	0.946	0.530

### Discussion

The current study is to describe radiation and time characteristics during mechanical thrombectomy in AIS patients. We also indicated that stroke severity didn’t affect these parameters.

Ischemic stroke is a leading cause of disability in the world and ranks as the fifth most common cause of death in US. [9,10] Endovascular therapy arises as an effective measure to save the devastating condition. [11,12] Intra-arterial thrombolysis facilitates artery recanalization and long-term functional recovery. [13] Mechanical thrombectomy is usually applied for large artery occlusion, with an intra-arterial catheter-guided technique under fluoroscopy. [14] A systematic review and meta-analysis in 2015 of randomized trials concluded that endovascular therapy significantly improved the clinical prognosis in patients with AIS compared with conventional treatment. [15]

Radiation produced during X-ray assisted artery angiography [16] may cause damage to human body. [17,18] The potential for malignant and skin diseases increases with the cumulative radiation exposure. [5] The modulation of peri-procedural radiation helps protect from exposure damage. Dose parameters are often used to estimate radiation risk. [19] The study recorded dose produced in all shots, fluoroscopy and cumulative to give a comprehensive understanding of radiation effects. For time parameter analysis, operation time covered both radiation exposure and other process, including patient transfer, catheter insertion. A recent study [20] indicated that male gender, number of passages and successful racanalization are independent key parameters affecting dose. However, we didn’t find significant change when analyzing based on gender. This needed further trials to demonstrate this point. Number of passages and successful recanalization were not recorded in this study. This could be analyzed in future study with more patients.

Large artery occlusion stroke intends to get into a severe neurological impairment status. Mechanical thrombectomy could effectively reversed it. All cases included in this study were affected in large cerebral artery and they received mechanical thrombectomy. This is an on-going prospective trial investigating the radiation effects in endovascular treatment for acute ischemic stroke. The current study was to summarize the available data to add our knowledge on this field. We would like to enroll more subjects in the future to demonstrate the effects of endovascular methods on procedure parameters.

Stroke patients in severe conditions sometimes were unable to cooperate with the examination. This thus might cause a prolonged operation and radiation exposure. We then measured the relationship between stroke severity and procedure parameters. NIHSS scoring system is a useful tool to measure stroke severity, which includes 15 items. However, no significance were found in the statistical analysis. This indicated that stroke severity dose not influence the time and dose during mechanical thrombectomy.

The current study provided the description of radiation and time parameters during mechanical thrombectomy in acute large artery occlusion. Stroke severity does not influence the radiation effects during the procedure. This added the understanding of radiation risk during interventional procedures.
